# Prescribing Trends and Associated Outcomes of Antiepileptic Drugs and Other Psychotropic Medications in US Nursing Homes: Proposal for a Mixed Methods Investigation

**DOI:** 10.2196/64446

**Published:** 2024-09-19

**Authors:** Jonathan D Winter, J William Kerns, Nicole Brandt, Linda Wastila, Danya Qato, Roy T Sabo, Stephen Petterson, YoonKyung Chung, Sarah Reves, Christopher Winter, Katherine M Winter, Eposi Elonge, Craig Ewasiuk, Yu-Hua Fu, Adam Funk, Alex Krist, Rebecca Etz

**Affiliations:** 1 Department of Family Medicine and Population Health School of Medicine Viginia Commonwealth University Richmond, VA United States; 2 Peter Lamy Center on Drug Therapy and Aging School of Pharmacy University of Maryland Baltimore, MD United States; 3 Department of Biostatistics School of Population Health Virginia Commonwealth University Richmond, VA United States; 4 Harvey L. Neiman Health Policy Institute Reston, VA United States; 5 Larry A. Green Center Richmond, VA United States

**Keywords:** Alzheimer disease, dementia, antiepileptic drug, antiseizure medication, antipsychotic, National Partnership, nursing home, mood stabilizer, COVID-19

## Abstract

**Background:**

Pilot data suggest that off-label, unmonitored antiepileptic drug prescribing for behavioral and psychological symptoms of dementia is increasing, replacing other psychotropic medications targeted by purposeful reduction efforts. This trend accelerated during the COVID-19 pandemic. Although adverse outcomes related to this trend remain unknown, preliminary results hint that harms may be increasing and concentrated in vulnerable populations.

**Objective:**

Using a mixed methods approach including both a retrospective secondary data analysis and a national clinician survey, this study aims to describe appropriate and potentially inappropriate antiepileptic and other psychoactive drug prescribing in US nursing homes (NHs), characteristics and patient-oriented outcomes associated with this prescribing, and how these phenomena may be changing under the combined stressors of the COVID-19 pandemic and the pressure of reduction initiatives.

**Methods:**

To accomplish the objective, resident-level, mixed-effects regression models and interrupted time-series analyses will draw on cohort elements linked at an individual level from the Centers for Medicare and Medicaid Services’ (CMS) Minimum Data Set, Medicare Part D, Medicare Provider Analysis and Review, and Outpatient and Public Use Files. Quarterly cohorts of NH residents (2009-2021) will incorporate individual-level data, including demographics; health status; disease variables; psychotropic medication claims; comprehensive NH health outcomes; hospital and emergency department adverse events; and NH details, including staffing resources and COVID-19 statistics. To help explain and validate findings, we will conduct a national qualitative survey of NH prescribers regarding their knowledge and beliefs surrounding changing approaches to dementia care and associated outcomes.

**Results:**

Funding was obtained in September 2022. Institutional review board exemption approval was obtained in January 2023. The CMS Data Use Agreement was submitted in May 2023 and signed in March 2024. Data access was obtained in June 2024. Cohort creation is anticipated by January 2025, with crosswalks finalized by July 2025. The first survey was fielded in October 2023 and published in July 2024. The second survey was fielded in March 2024. The results are in review as of July 2024. Iterative survey cycles will continue biannually until December 2026. Multidisciplinary dissemination of survey analysis results began in July 2023, and dissemination of secondary data findings is anticipated to begin January 2025. These processes are ongoing, with investigation to wrap up by June 2027.

**Conclusions:**

This study will detail appropriate and inappropriate antiepileptic drug use and related outcomes in NHs and describe disparities in long-stay subpopulations treated or not treated with psychotropics. It will delineate the impact of the pandemic in combination with national policies on dementia management and outcomes. We believe this mixed methods approach, including processes that link multiple CMS data sets at an individual level and survey-relevant stakeholders, can be replicated and applied to evaluate a variety of patient-oriented questions in diverse clinical populations.

**International Registered Report Identifier (IRRID):**

DERR1-10.2196/64446

## Introduction

### Background

Despite safety concerns and limited evidence of efficacy, antipsychotics have been highly used to treat behavioral and psychological symptoms of dementia (BPSD), particularly in nursing home residents [1,2]. Due to their risk profile and high prevalence of use, antipsychotics have also been singled out for medication reduction efforts. The Centers for Medicare and Medicaid Services (CMS), the Food and Drug Administration (FDA), patient advocacy groups, and others have taken significant steps to curb inappropriate antipsychotic use in vulnerable older adults [3-5]. In 2012, the CMS debuted the *National Partnership to Improve Dementia Care in Nursing Homes*, an initiative that included targeted efforts to reduce antipsychotic use among nursing home residents with Alzheimer disease (AD) or AD-related dementias (ADRD) [1]. Subsequently, the percentage of long-stay residents who receive antipsychotic medication has been measured and graded as a long-stay quality measure in the Minimum Data Set (MDS) [6].

Since the National Partnership, antipsychotic use in nursing homes has decreased substantially [7-9]. However, this and other regulatory efforts to curb antipsychotic use have resulted in unintended outcomes, including increased prescribing of alternative, potentially high-risk agents [10]. For example, data suggest increased use of mood-stabilizing antiepileptic drugs (AEDs), which are not monitored by the CMS or assessed using a quality measure [11-15]. All AEDs have the potential for severe side effects, particularly for older adults, and several mood-stabilizing AEDs carry *black box* warnings for use in older patients [16-18]. Exactly how AEDs are prescribed in US nursing homes is unknown, as, unlike other psychotropics, a detailed national assessment has not been reported for almost 20 years [19-25]. In 2018, Maust et al [12] described the prevalence of psychotropic classes in nursing homes from 2009 to 2014 and reported a decline in long-stay prescribing of all classes of psychotropics except mood-stabilizing AEDs, which accelerated after the 2012 National Partnership debut. Other studies support this finding of increased mood-stabilizing AED use in nursing homes, with the greatest increases seen in residents without seizure epilepsy [11,12,14,15,23,26-33]. In addition to the unintended prescription shift in response to the National Partnership’s initiative, analysis of Virginia claims data suggests that the pandemic is driving a second increase in AED prescribing, leading to a profound effect on dementia care and nursing home outcomes [14,15,31-34]. As AEDs are the one class of psychotropic medications not mandatorily reported in the MDS, prescribing changes would go undetected by the CMS and other stakeholders in dementia care.

With the goal of increasing understanding regarding the use of all psychoactive medications in nursing homes, we began a mixed methods investigation deploying both quantitative and qualitative approaches to describe how and why AEDs and other psychotropics are used in nursing homes; the characteristics and outcomes associated with their use; and how their use may be changing as the COVID-19 pandemic stresses long-stay facilities, unmasking existing deficiencies and gaps in care. Guided by quality improvement frameworks, we hypothesize that, as antipsychotics and anxiolytics are used less in nursing homes, prescribing of mood-stabilizing AEDs as unmonitored alternatives has increased. To test this general hypothesis, we will be guided by the study aims outlined in the following sections.

### Specific Aims

#### Aim 1

##### Overview

The first aim is to evaluate the prevalence; use trends; and associated patient, facility, and regional characteristics of AEDs and other psychotropics prescribed to nursing home residents, including those with AD and ADRD.

##### Subaim 1a

The first subaim of aim 1 is to measure the quarterly prevalence of individual AEDs used in nursing homes (2009-2021) and investigate changes in prevalence trends before and during the COVID-19 pandemic and surrounding the introduction of the National Partnership among all long-stay residents, focusing on those with AD and ADRD*.*

##### Subaim 1b

The second subaim of aim 1 is to evaluate appropriate and potentially inappropriate AED prescribing, including for AD and ADRD (2009-2021), and investigate changes surrounding the pandemic and the National Partnership’s debut.

##### Subaim 1c

The third subaim of aim 1 is to compare the patient (demographic, medical, and cognitive) characteristics, facility elements, and regional variables of US nursing home residents prescribed or not prescribed AEDs in 2021.

##### Hypothesis 1

The potentially inappropriate long-stay use of mood-stabilizing AEDs for AD and ADRD is increasing as other psychotropic drugs are used less, a trend that accelerated during the COVID-19 pandemic, with vulnerable populations from less resourced facilities being at greatest risk of unsafe prescribing.

#### Aim 2

##### Overview

The second aim is to evaluate the association between adverse health events and individual AED and other psychotropic prescribing (2009-2021) for all indications, including AD and ADRD, and investigate changes surrounding the COVID-19 pandemic and the National Partnership’s debut, adjusting for patient, facility, and regional variables.

##### Subaim 2a

The first subaim of aim 2 is to evaluate the association between individual AED prescribing and adverse health events, including detrimental nursing home outcomes (ie, falls; decreases in cognitive, functional, mood, and behavior scores; hospice or palliative needs; and death), hospitalizations, and emergency department (ED) visits.

##### Subaim 2b

The second subaim of aim 2 is to compare the patient characteristics (including AD and ADRD), facility elements, and regional variables of nursing home residents with and without adverse prescribing complications.

##### Subaim 2c

The third subaim is to investigate trends in the quarterly rates of adverse health events, including detrimental long-stay outcomes, ED visits, and hospitalizations of residents (concentrating on AD and ADRD) prescribed or not prescribed AEDs (2009-2021), focusing on prevalence changes surrounding the pandemic and the National Partnership’s debut.

##### Hypothesis 2

Adverse prescribing complications are associated with individual AED use, which increased during the COVID-19 pandemic, and are more common among residents who have AD or ADRD and are frail, Black and African American or Hispanic, and from facilities and regions with fewer resources.

#### Aim 3

##### Overview

The third aim is to develop and implement a survey based on the theory of planned behavior to assess the knowledge, attitudes, and beliefs of US long-stay prescribers regarding their current and evolving approaches to managing AD and ADRD and other dementias, with an emphasis on outcomes and the impact of policies and the pandemic.

##### Hypothesis 3

National data from purposefully sampled nursing home clinicians can affirm the validity of all study findings, support the understanding of mechanisms underlying prescribing patterns, and elaborate on prescribing outcomes while identifying critical opportunities to mitigate disparities and improve AD and ADRD care and safety.

### Clinical Implications

This study will address clinically meaningful evidence gaps directly relevant to clinical guidelines and policies and is consequential to all stakeholders in dementia care, including residents, providers, specialty groups, payers, patient advocates, and policy makers. If pharmacological approaches to BPSD management rapidly evolved during the pandemic, with risky and ineffective—but unreported—medications being used more, then harms could increase with no clinical or safety benefit, jeopardizing the health and safety of all nursing home residents, especially vulnerable ones. We know very little about outcomes or adverse health events associated with this changing approach to dementia care. Our proposed study will close this existing knowledge gap by providing a clearer picture of all long-stay pharmacological approaches to noncognitive dementia symptoms, the health outcomes associated with these varying approaches, and how such management and consequent outcomes are changing under the pressure of pandemic stressors and targeted medication reduction efforts.

## Methods

### Overview

We propose a mixed methods approach including both a retrospective secondary data analysis and a national survey to describe appropriate and potentially inappropriate antiepileptic and other psychoactive drug prescribing in US nursing homes, characteristics and patient-oriented outcomes associated with this prescribing, and how these phenomena may be changing under the combined stressors of the COVID-19 pandemic and the pressure of reduction initiatives.

### Secondary Data Analysis

#### Data Sources and Data Management

We will create a combined data set linking elements extracted from multiple CMS files that will collectively allow for a quarterly assessment of the use rate of individual AEDs and other psychotropic medications in US long-stay facilities over a 12-year study period as well as the nursing home, ED, and hospital adverse outcomes associated with their use while controlling for important demographic, health, regional, and facility variables. This period will encompass the 3 years before and 9 years after the National Partnership’s 2012 debut, as well as the 10 years leading up to and the first 2 years of the COVID-19 pandemic. Thus, trends in prescribing and outcomes associated with prescribing will be assessed with an emphasis on evaluation for change surrounding 2 primary inflection points: the March 2012 debut of the National Partnership and the January 2020 commencement of the COVID-19 pandemic [35-44].

This study includes data elements extracted from 5 sources: the CMS MDS, the Medicare Part D Drug Event File, the Medicare Provider Analysis and Review (MedPAR), and 2 CMS public files. The CMS *Long-Term Care* *MDS 3.0* is a systematic assessment of the health of nursing home residents performed at admission, annually, and—for a subset of items—quarterly or when the resident has a change in health status [6,35,38,45]. The MDS will be used to define the study cohorts by identifying all US long-stay residents during the period of January 2009 to December 2021 and contains patient identifiers and demographic and regional variables, as well as a wealth of quarterly data on medication use (not including AEDs); chronic health conditions; and updated cognitive, psycho-behavioral, functional, and prognostic outcome data.

The *Medicare Part D Drug Event File* provides prescription fill information, including drug name, National Drug Codes, date of dispensing, days’ supply, and dosage for all beneficiary medications paid for under the Medicare Part D Prescription Drug Program. AED prescription fill information for 2009 to 2021 will be extracted [39,46].

The *Provider Information Public File* available at data.cms.gov [47] provides quarterly quality data for all long-stay facilities, including the quality metrics that inform the CMS’s 5-star facility rating and that are available for public review [11]. These data can be accessed at the Nursing Home Compare data archive beginning from July 2013 [36]. Variables to be extracted include facility variables such as ownership type and size, various facility ratings, and staffing details.

For facility COVID-19–related data, we will use the CMS’s *COVID-19 Public File* [42]. This file, sourced from the Centers for Disease Control and Prevention National Healthcare Safety Network system Long-term Care Facility COVID-19 Module, includes weekly data elements (first reported on May 17, 2020, but entered retroactively up to January 1, 2020) describing facility COVID-19 status, facility capacity, and staffing or personal protective equipment adequacy.

Finally, for hospital and ED data, we will incorporate individual-level results from the *MedPAR* and *Outpatient Research Identifiable File* (RIF). The MedPAR file was specifically created by the CMS for the study of inpatient hospital care and contains claims for all Medicare Part A inpatient stays [40]. MedPAR Medicare Advantage claims are incomplete (with no data available from 2009 to 2014), and consequently, only patients insured with Medicare Fee-For-Service will be included in all portions of the analysis. However, we will request Medicare Advantage inpatient and outpatient data for the years that are available (2015-2021), and we will incorporate these findings into the analysis where possible. The Outpatient file includes Fee-For-Service claims associated with outpatient hospital care. This includes all claims submitted by institutional outpatient providers, including outpatient hospital care. Using MedPAR and Outpatient RIFs, we will identify ED visits, hospitalizations, hospitalization diagnoses (including COVID-19), length of stay, and hospitalization costs. Beneficiary identifiers (namely BENE_ID) extracted from the MDS will be used as crosswalks to link MDS, MedPAR, Outpatient, and Medicare Part D files at an individual level. The MDS–Part D link has been negotiated successfully by multiple other investigators with matching errors of 4% to <1% [48,49]. The MDS-MedPAR link is more challenging as it requires both a BENE_ID file match and a temporal match where MDS Part A transition dates are linked with MedPAR data for that same period. For this link, a match rate approaching 90% is expected [50,51]. Identifying ED visit claims is more straightforward as no MDS transition is involved and is accomplished simply by searching for a MedPAR or Outpatient ED charge amount of >0 for that quarter. National Provider Identifiers will serve as crosswalks between individual-level data from the MDS, Part D, MedPAR, and Outpatient files and the facility-level data from the 2 facility files. Data will be compiled in the CMS’s Virtual Research Data Center (VRDC) and accessed via a Data Use Agreement (DUA) with the CMS [41]. The CMS’s VRDC is a virtual research environment that allows access to CMS data in a more secure, timely, and affordable manner [41]. All person-level health information will remain in the virtual data center for analysis, a virtual workspace that meets all CMS safety and security requirements. Only deidentified data downloads are eligible.

#### Final Cohort Construction

Long-stay residents with >100-day stays at a nursing home facility will be identified from the CMS MDS (2.0 for 2009-2010 and 3.0 for 2010-2021). The timing and location of stay will be confirmed based on a previously validated residential history file algorithm [43]. Cohorts will be limited to residents with continuous Fee-For-Service or Medicare Advantage plans as well as continuous Part D coverage over that quarter and those who are aged >21 years [44]. Research Data Assistance Center enrollment files will allow us to determine beneficiaries’ continuous enrollment. Nursing home residents who are discharged before the end of the quarterly study period will be excluded starting from the quarter in which discharge occurred. Residents with discharges for acute hospitalizations followed by facility re-entry on the same record will not be excluded. A total of 48 quarterly cohorts comprising all nursing home residents will be identified for the 12-year period of January 2009 to December 2021. Quarterly analysis is ideal as this is how MDS data are collected and organized.

#### Data Linkage and Data Collection

Once cohorts are created, the following information will be extracted from the MDS: resident and facility identifiers, demographic data, regional data (using facility identifier and geolocation), and resident-specific health variables that are mandatorily reported by nursing home facilities to the MDS. Beneficiary identifiers will be used as crosswalks to query Part D claims for at least one fill of an AED prescription in that quarter, and medication use will be assessed and organized at a quarterly level [46]. Claim date and days of supply will allow drug use to be attributed to all appropriate quarters (for example, a 2-week fill in a quarter’s last week will count for both quarters). AEDs will be identified by name (generic and brand) as well as by National Drug Codes (9, 10, and 11) from Part D claims and further mapped to the appropriate drug using drug databases such as Multum. Lithium and dextromethorphan or quinidine (Nuedexta) use will also be evaluated as they affect mood. All other psychotropic medications, including antipsychotics, anxiolytics, antidepressants, and sedative-hypnotics, will be similarly queried from Part D and at a class level from the MDS and reported as MDS, Part D, and MDS–Part D. Comparing data from different classes and sources will serve a function in quality control. Similarly, as some AEDs are prescribed for pain, opiates will be analogously assessed.

To add expanded facility data to our growing cohort, we will incorporate data from 2 publicly available long-stay facility files. The first is CMS’s Provider Information file that collates all the quarterly facility quality data that inform the CMS’s 5-star facility ratings [52]. Variables to be extracted include ownership type (for profit and not for profit), facility size, star rating, and staffing rating, as well as aide, licensed professional nurse, registered nurse, and therapist staffing hours. All staffing hours will be adjusted per resident day and also resident case-mix index. For COVID-19–related facility data, we will use the COVID-19 Public File [42]. This public file comprises data reported by nursing homes to the Centers for Disease Control and Prevention National Healthcare Safety Network system Long-term Care Facility COVID-19 Module. This data set includes weekly data elements (first reported on May 17, 2020, but entered retroactively up to January 1, 2020) describing facility COVID-19 status and facility capacity as well as staffing, supplies, and personal protective equipment adequacy. Monthly and weekly data from the Provider Information and COVID-19 Public Files will be summed or averaged to correspond with quarterly cohort data. Where weeks or months do not match quarters, data will be proportionately divided.

Finally, for hospital and ED outcomes, we will incorporate data at an individual level from MedPAR and Outpatient RIFs. Hospitalization dates reported in section A of the MDS will be linked with inpatient data extracted from MedPAR for that same period. Hospital data to be extracted from MedPAR include hospital admission and discharge date, principal diagnosis codes, death, and hospital costs. In addition, admission diagnoses can be used to identify both “potentially avoidable” and “medication-related” hospitalizations using previously validated algorithms. Both protocols were developed by expert panels and include detailed International Classification of Diseases, 9th and 10th Revision, codes [53-57]. Thus, “potentially avoidable” hospitalizations, “medication-related” hospitalizations, and hospitalizations overall can be compared between cohorts treated or not treated with a psychotropic and across medication types. ED data to be extracted from MedPAR and Outpatient files include ED charge amount and ED-related revenue center codes to determine the ED visit rate between the aforementioned cohorts.

### Survey Assessment

#### Overview

The *Psychotropic Prescribing in Nursing Homes* (PPIN) survey goals are to confirm and validate the findings of aims 1 and 2 while adding context, detail, meaning, and clarity to their results. We will survey a purposive national sample of nursing home psychotropic prescribers regarding their use of all psychotropics with an emphasis on the impact of reduction efforts and the pandemic on their current and historic prescribing practices. This will be accomplished by (1) drawing on an existing multidisciplinary team experienced in national clinician surveys and mixed methods analyses of long-stay issues, (2) using existing pilot study data to inform initial survey development, and (3) using the theory of planned behavior to guide design and analysis [58-61].

#### Conceptual Framework

Construction and analysis of the PPIN survey is based on the theory of planned behavior by Azjen [60]. The theory of planned behavior outlines the relationship among beliefs, attitudes, norms, and human behavior; is a well-supported model for examining human behavior; and has been effectively applied specifically to surveys on health care–related decisions [60-63].

#### Survey Content

Due to the unprecedented turbulence in long-term care, survey construction will follow an adaptive sequential exploratory design process. Accordingly, the PPIN survey will include 6 survey cycles over a 36-month period to allow for flexibility in exploring relevant themes and issues perhaps currently unforeseen. While the initial survey will be grounded in existing literature and our own pilot data, each iterative version will be informed by retrospective and emergent data, including early data generated by aims 1 and 2 [15,31]. Early survey results may also provide direction to the ongoing investigations of aims 1 and 2. Given the ongoing and unprecedented period of dynamic stress in long-term care related to the COVID-19 pandemic, assuming transformation over the survey period is both reasonable and potentially clinically meaningful. For the same reason, it would not be appropriate to develop all survey instruments before the start of the study—the ideal survey today will likely be obsolete by the time of fielding. The PPIN surveys will include 4 key components: “Core” questions, “Flash” questions, demographic questions, and open-ended comments. Core questions will consistently assess issues surrounding the decision to prescribe or not prescribe psychotropic medications, including AEDs, in nursing homes, with a focus on prescribing for dementia symptoms. We will specifically explore the impact of reduction measures and the COVID-19 pandemic on management approaches and outcomes. This will include questions detailing factors that encourage or discourage the use of medications and encourage or discourage the use of nonpharmacologic therapy (NPT) for psychiatric symptoms. “Flash” questions are so named for the way in which they mimic flash mobs—sudden, impactful, meaningful, and then gone. The flash questions will allow the research team to be adaptive and iterative through the survey process, providing the opportunity to explore new or unanticipated issues, including data deficiency. Every cycle will allow participants at least one opportunity for comments with open-ended questions, at times expanding to longer open opportunities. These responses will inform future flash questions. While flash questions will vary each cycle, thematic consistency still allows for longitudinal qualitative analysis. Finally, all surveys will include demographic questions about the prescriber and prescriber location to allow for subanalyses of findings.

#### Survey Development and Sampling Frame

This survey will be designed to minimize respondent burden, asking only the most relevant pieces of information each cycle, with a goal of a 4- to 5-minute response time. We will spend the first year after funding finalizing a pilot survey informed by initial quantitative findings. Initial questions and response options will be developed from our published and unpublished previous work and relevant literature [15,31]. Pilot questions will be tested using cognitive interviews, an approach that this team has used successfully in past studies [64,65]. Once the initial survey has thus been generated, we will field our first survey cycle at the 12-month mark to obtain a baseline. This means that our baseline survey will be unbiased by the preliminary results of aims 1 and 2 but would be timely in informing the ongoing evaluations of aims 1 and 2*.* A total of 6 biannual survey cycles will continue over a 36-month sampling frame. We anticipate preliminary data from aims 1 and 2 to become available by 18 months. Survey content will be updated each cycle based on previous surveys and early data from aims 1 and 2. The survey advisory committee—a multidisciplinary subgroup of the research team primarily dedicated to the successful completion of aim 3—will vet questions for each cycle, iterating proposed response options and scales and modifying survey focus. The goal will be to improve clarity with each cycle while allowing for the potential to explore unexpected new avenues or add granular detail to an area deserving further clarification. The committee, survey respondents, and survey partners will all have the opportunity to submit potential new questions. The final survey is selected by the advisory committee. This stakeholder-engaged process is based on lessons learned from previous National Institutes of Health– and Agency For Healthcare Research and Quality–funded studies conducted by our investigative team [65-70]. This iterative approach will be of value over this dynamic period in health care in which the perfect survey instrument for today could well be incomplete tomorrow.

#### Participant Recruitment and Survey Distribution

Participants will be licensed nursing home clinicians who prescribe psychotropic medications, including advanced practice clinicians and physicians. Our goal is a sample of >500 participants per cycle, purposefully selected to reflect what is known about the national long-stay psychotropic prescriber population. Given that the intent of this investigation is not to create knowledge or test hypotheses but rather to confirm and validate the findings of aims 1 and 2 while outlining the forces and influences driving them, an exact representative sample is not required; a purposive sample will be sufficient to accomplish our aims [71]. By *purposive*, we mean a sample that is deliberately recruited to reflect the little that is known about the regional, demographic, and professional characteristics of the national long-stay prescribing population. We will accomplish this by collaborating with partner professional organizations whose membership are known to prescribe psychotropics in nursing homes in a direct emailing campaign [44,72,73]. Data will be collected using SurveyMonkey. Partnering organizations will receive “partner emails” that include priority requests from our research team (eg, a need to increase participation among minority groups and rural areas) and easy-to-use social media information to enable quick sharing of the survey link via organization-driven social media. These organizations may choose to share the SurveyMonkey survey link with their membership via email or advertise the SurveyMonkey survey link to membership on their social media platforms. Alphanumeric codes embedded in the survey link will allow our team to track a longitudinal cohort while maintaining the anonymity of participants at the point of data collection.

#### Survey Responses

Our understanding of exactly who prescribes psychotropics in US nursing homes is imperfect [25,73-76]. We estimate the total number to exceed 100,000 clinicians, with most being internists, family physicians, and nurse practitioners and a minority being psychiatrists, neurologists, and physician assistants. On the basis of our recent and ongoing experience collaborating with professional organizations on electronic clinician surveys, we anticipate an initial survey distribution to 10,000 clinicians. Our historical survey response rate from clinicians when recruiting in partnership with professional organizations has been approximately 5% [65,68,69]. Thus, we anticipate a sample of >500 participants for each cycle. We accomplished our published response rates by “advertising” our survey with links on social media, including postings by partner organizations on partner organization websites and the use of the modified Dillman method [77]. We are successfully using near-identical methodology for surveying clinicians in an ongoing Agency For Healthcare Research and Quality R01–funded investigation, and lessons learned will inform this investigation [65-67,78] However, while 2 years of data collection and 35,000 web-based surveys received engenders confidence in this methodology, we acknowledge a unique practice environment during the pandemic where it may be prudent to anticipate response rates lower than in the past, so we have budgeted for the possibility of purchasing email information for long-term care prescribers from commercial vendors. These data will be purposefully selected based on regional, demographic, and educational clinician characteristics. We have a proven track record with this approach, and while it is more labor intensive and expensive, we have reached response rates approaching 30% and are comfortable with methodology including protocols to protect participant anonymity [79].

### Outcomes

#### Aim 1

The primary outcome of aim 1 will be quarterly use rates of each AED and other psychotropics in the general study population for the entirety of our study period. A secondary outcome will be quarterly use rates of individual AEDs among specific subsets of long-stay residents. AED prescribing within these subsets can then be compared. Subsets of long-stay residents will be those with and without dementia, seizure epilepsy, psychiatric illness, and neuropathic pain. A final subset will be those with and without an appropriate diagnosis to support the specific AED prescribed. As Medicare Part D does not link prescriptions to their indication, we will approximate the appropriateness of prescribing by identifying the presence or absence of FDA-approved diagnoses in the MDS data of the recipient to support the specific AED used. If an approved diagnosis for a given AED exists, that prescription will be approximated as appropriate. However, if no approved diagnosis exists, that prescription will be approximated as inappropriate. For all analyses, the rate numerator will be the number of individuals within a cohort filling at least one psychotropic prescription during that quarter. The rate denominator will be the quarterly cohort, or subcohort, of all long-stay residents meeting the inclusion criteria.

#### Aim 2

The primary outcome of aim 2 will be the quarterly rate of adverse health events among long-stay residents prescribed or not prescribed individual AEDs and other psychotropics. Adverse health events to be evaluated include several adverse nursing home outcomes as well as ED and hospital outcomes. Rate numerators will be the relevant nursing home outcome reported for that quarter or the presence of an ED or hospital event at any time during that quarter. Rate denominators will be the quarterly cohort of long-stay residents receiving the pertinent AED or other psychotropic at any time during that quarter versus those not receiving it. In the analyses for both aims, we will control for demographic and regional variables and functional, health, and disease markers, as well as facility data, including ownership, performance scores, staffing, and COVID-19–related information.

#### Aim 3

The primary outcomes of aim 3 will be diverse. First, they will serve to affirm and validate the results of aims 1 and 2 while informing the extractions and analyses of the secondary data assessment. In addition, survey results should clarify which drugs are used or not used for dementia symptoms and why. They should outline impacts of policy efforts on prescribing and prescribing outcomes and present the impact of the COVID-19 pandemic on prescribing and prescribing outcomes. Results will also contrast management by prescriber, facility, and regional variables. Finally, they should delineate barriers to the use of nondrug therapies for dementia symptoms.

### Statistical Analysis

#### Subaim 1a

The 2021 prevalence of individual AEDs and other individual psychotropic medications in US nursing homes will be estimated and compared by geographic regions using a mixed-effects logistic regression model with a 4-level fixed-effects quarterly time indicator and both patient-level and nursing home–level random effects to account for within-subject dependence and the nesting of patients within nursing homes. Differences in the trends of AED prevalence over the 12-year period from 2009 to 2021 will be investigated using a patient-level interrupted time-series analysis [80]. AED and other psychotropic medication prevalence will be modeled individually as binary outcomes (prescribed in a particular quarter or not) against a continuous time trend (both linear and quadratic), indicators for (1) pre– and post–National Partnership and (2) pre– and post–COVID-19 period, and an interaction between time trend and period (significant indicator or interaction effects are indicative of differential trends between periods) [80]. A model will be used to identify temporal changes: one occurring before or after March 2012, reflecting potential reactions to the National Partnership guidance change, and the second occurring before or after January 2020, reflecting potential changes due to COVID-19. This model will also account for within-patient repeated measures through an auto-regressive correlation structure as residents are continuously enrolled and prescriptions are assessed evenly (quarterly) in the study databases. A facility-level random effect to account for nesting of patients within nursing homes will also be included. While we hypothesize 2 primary inflection points (the National Partnership and COVID-19), due to the potential for other impact points, we will conduct sensitivity analyses looking for secular trends in our data to identify the possibility of other policy inflection points.

#### Subaim 1b

To compare the 2021 prevalence of individual AEDs between subsets of nursing home residents with and without a diagnosis approximated as appropriate, we will expand on the logistic regression model described in the *Subaim 1a* section to include only the long-stay subset. The odds of AED prevalence (and their 95% CI) will be estimated in each of the 4 quarters in 2021. Trends of quarterly AED prevalence from 2009 to 2021 will be compared between subsets of nursing home residents with and without a diagnosis approximated as appropriate by extending the interrupted time-series model described for subaim 1a to include interactions between time and each of these terms modeled using a binary indicator [81].

#### Subaim 1c

Resident (demographic, medical, and functional) characteristics, facility elements (resources and ownership status), and regional variables will be compared between those US nursing home residents prescribed individual AEDs or other psychotropics at any point in 2021 and those who were not prescribed such drugs using linear and generalized linear mixed-effects models, including fixed effects for quarter (4 levels), AED prescription indicator (yes or no), an interaction term, and both patient-level and site-level random effects.

#### Subaim 2a

The association between individual AED and other psychotropic prescribing and adverse health events, including nursing home outcomes, hospitalizations, and ED visits, in 2021 will be assessed using multiple linear or logistic mixed-effects models controlling for resident, facility, and regional variables. Fixed effects in these models will include binary prescription indicators, a 4-level quarter indicator, their interaction, and control variables. Patient-level and site-level random effects will be included to account for within-patient dependence and the nesting of patients within nursing homes, respectively.

#### Subaim 2b

Resident (demographic, medical, and functional) characteristics, facility elements (resources and ownership status), and regional variables will be compared between those US nursing home residents with adverse prescribing complications from AEDs and other psychotropics in 2021 and those who were prescribed such drugs without adverse prescribing complications using linear and generalized linear mixed-effects models, including fixed effects for quarter (4 levels), an adverse event indicator (yes or no), an interaction term, and both patient-level and site-level random effects.

#### Subaim 2c

Differences in the quarterly prevalence of adverse health events (including detrimental long-stay outcomes, ED visits, and hospitalizations) among nursing home residents prescribed or not prescribed AEDs or other psychotropics over the 12-year period of 2009 to 2021 will be determined using interrupted time-series models (similar to those described for subaims 1a and 1b), with the binary adverse health event measure included as the longitudinal outcome modeled against a binary AED prescription indicator, continuous time trends, and their interaction; within-resident dependence will again be modeled using a first-order auto-regressive structure, and a site-level random effect will be included to account for patient nesting within nursing homes.

#### Aim 3

Quantitative and qualitative analyses are first conducted separately and then combined. Structured data will be described using summary statistics (means and SDs for numerical responses and frequencies and proportions for categorical responses). Univariate statistical tests (1- and 2-tailed *t* tests, ANOVAs, and chi-square tests) will be used to assess whether the observed changes are due to chance variation or are likely to be real. Subgroup analyses will be performed comparing outcomes by provider type; facility variables; and regional variables, including social determinants of health for that location. A dedicated division of the research team will read through open-text comments for each survey cycle to identify main content areas. This, combined with elements of the theory of planned behavior and our previous research, will be used to create an a priori codebook to guide a template-based analysis [60]. Analysis will incorporate both template-based and emergent coding techniques. We will follow a protocol-driven approach to analysis that involves 3 steps. The first step involves group reading of the data to refine a priori codes, identify emergent codes, and reach agreement on code definition. Second, independent test coding is performed to probe the operational limits of the codebook and the ability of coders to apply codes reliably and consistently. Finally, independent coding combined with scheduled merges of coded data and monthly team coding huddles allow for early detection of threats to intercoder reliability [82]. Once coded, the team identifies themes within the data. All free-response qualitative data will be managed using ATLAS.ti (ATLAS.ti Scientific Software Development GmbH) [83].

### Sample Size Determination

Our study’s power to detect effect differences in AED prescribing trends over time assumes 1,406,220 nursing home residents in the United States; that nearly 14% and 38% of nursing home residents are diagnosed with epilepsy or dementia, respectively; and that 4.5% of nursing home residents are prescribed mood-stabilizing AEDs [7,33,44,84]. Assuming a quarterly AED prevalence near 4.5% with 1.4 million nursing home residents will allow for a margin of error of <0.1% (subaim 1a). On the basis of these quarterly estimates over 12 years (48 total estimates), we will have 80% power to detect differences in the rates of change in AED prevalence between periods as small as 0.52%*,* which is similar to the decrease in rates of psychotropic or memory medications observed in the literature. Similar power and detectability can be expected for the comparative analyses in subaims 1b, 1c, 2a, 2b, and 2c. For aim 3, quantitative analyses include a comparative time study evaluating changes over the 36-month sampling window. We hypothesize that the differences observed for most clinician outcomes will fall in the large effect size category. Still, with estimated sample sizes of 1000 clinicians per period, our sample sizes are sufficiently large to detect even small effect sizes [85]. For analyses of outcomes, a planned sample size of 1000 residents in each comparison time has 90% power to detect a standardized effect size of 0.14 with a significance level of 0.05 [86]. For quantitative outcomes, an effect size of 0.14 would be considered very small as defined by the Cohen *d* statistic [85]. Samples of 500 clinicians per period would also enable the detection of a comparable small difference in outcomes between periods. While the clinician cross-sectional samples can be expected to be independent samples, they may contain some overlap, thus introducing auto-correlation. To address this, our approach to significance testing between periods will be to conduct ordinary least squares statistics (eg, *t* test and ANOVA) and then modify the SEs afterward [87]. Subgroup analyses will be based on at least a 20% sample of the total, and a sample of 200 clinicians will have 80% power to detect a standardized effect size of 0.2 at a 0.05 level of significance. Thus, even for the subgroup analyses, we will have adequate statistical power to detect small effect size differences [85].

### Ethical Considerations

Only deidentified aggregate data were available to the research team, and no participant received compensation. This study has been approved by the Virginia Commonwealth University (VCU) Institutional Review Board (IRB; HM20025382) and poses minimal risk to participants. The risks include breaches of confidentiality and privacy. Due to the retrospective nature, the protocol qualified for exemption and Health Insurance Portability and Accountability Act authorization. Consequently, the VCU ethics committee on the IRB waived the need for written informed consent from all participants or their legal guardians. All methods were carried out in accordance with relevant guidelines and regulations.

## Results

### Overview

Funding for this 60-month project was obtained in September 2022. The project mobilized with a pre–data extraction phase. The entire research team assembled for at least monthly meetings to evaluate goals and prepare for future project activities. The first critical task was to acquire IRB exemption approval. IRB approval was obtained in January 2023 (IRB HM20025382). In parallel to the IRB application, we developed our DUA with the CMS. Our proposal was submitted in May 2023. This went through 2 rounds of comments as well as an internal institutional review. The DUA (RSCH-2024-70173) was signed in March 2024.

### Secondary Data Analysis

In our DUA, we applied for regular researcher access to the Chronic Conditions Warehouse in the VRDC [41]. Access was obtained in July 2024. The VRDC facilitates more rapid and convenient access to our data sets of interest within a CMS-approved secure virtual workspace, which eliminates many of the logistical challenges of ensuring the safety and security of resident personal health information [36,41,88]. There are many sequential steps to preparing a DUA and obtaining VRDC access. The team obtained access to the VRDC 14 months after our DUA was first submitted. It took 9 months to develop our DUA, a process that included obtaining IRB approval.

We purchased 2 years’ worth of access to the VRDC using an Interagency Agreement Number assigned in October 2023. This 2-year period began in July 2024 at the start of year 3 of the investigation. The critical milestone for the first half of year 3 will be building our quarterly sample cohorts in the secure VRDC environment using patient-level data from the MDS [89]. The next critical milestone will be linking these MDS cohorts with pharmacy claims data from the Part D Drug Event File as well as ED and hospital data from MedPAR and Outpatient files [39,46]. Facility-level facility data, including COVID-19 statistics from CMS public files, will enrich the cohorts further [42,72]. Beneficiary identifiers will be used for patient-level data crosswalks. Constructing our multisource cohorts within the VRDC will be an intensive 12-month process. We anticipate that cohorts will be created and the linkage with Part D data will be completed by December 2024, the halfway point of year 3. The full data set will take an additional 6 months to complete.

Our cohorts will include all available data. MDS data are easily accessible beginning from 2011. Files are continually updated but may lag real time by as much as 12 months [38,39]. Notwithstanding, in 2024, we should have access to data up to 2023. Evaluating data for completeness and accuracy will be an ongoing task while we have VRDC access in years 3 and 4. Queries will be revised and resubmitted if necessary with technical support from Research Data Assistance Center personnel. Early analysis of team research questions will occur within the secure virtual environment of the VRDC beginning in the second quarter of year 3. Deidentified data extracted from the VRDC and eligible for formal analysis are anticipated to be available to the full team by the third quarter of year 3. These extractions will continue until our VRDC access expires at the end of year 4. The final 18 months of the investigation will focus on analysis of data and dissemination.

### Survey Assessment

Parallel to our CMS data request, we initiated the qualitative portion of our evaluation: the development, validation, distribution, and analysis of a series of questionnaires surveying the perspectives of long-term care facility psychotropic prescribers. The intent of this survey series will be to confirm and validate our quantitative findings while outlining the forces driving these outcomes, with an emphasis on policies and the impact of the ongoing COVID-19 pandemic. Our protocol calls for us to debut a new and iterative survey every 6 months for a 36-month sampling window (running from years 2 to 4) as part of a sequential exploratory design. Iterative survey content will be informed by early results of our CMS data request. Conversely, our data request may be modified by early survey results and feedback from early dissemination.

The first 12 months of the investigation were spent developing and validating the initial survey instrument. The first survey was fielded in October 2023; the second survey was fielded in March 2024. We anticipate that the third survey will debut in September 2024. Survey cycles will continue roughly every 6 months for a total of 6 cycles to be completed at the end of year 4. The first survey focused on indications for gabapentin prescribing and clinician beliefs and perspectives regarding gabapentin increases. The second survey emphasized the relative frequency of identified indications and the role of gabapentin deprescribing. The third survey will concentrate on issues related to long-stay valproate use; the fourth survey will focus on lamotrigine and carbamazepine derivatives. Early mixed methods analysis began immediately after the first survey cycle. These will continue throughout the survey window. Our timeline’s overlapping data collection, analysis, and dissemination or feedback seeking reflects the iterative nature of our methods. Analysis and the first stages of dissemination began well before the project’s midpoint. Formal survey analyses were initiated as soon as the first survey results became available in year 2. Analysis will be ongoing until the end of the 5-year study. Multimodal and multidisciplinary dissemination and knowledge translation was introduced in year 2 as well and, similarly, will also continue throughout the study period. The results of our first survey were published in July 2024 [90]. We anticipate publication of findings of the second survey before the end of the year 2024.

## Discussion

### Expected Findings

While federally mandated reporting of antipsychotics, anxiolytics, antidepressants, and sedative-hypnotics to the MDS has helped clarify the use of most psychoactive medications in nursing homes, a clinically meaningful knowledge gap still exists regarding the long-stay use of unmonitored mood-stabilizing AEDs [91]. Over the last 10 years, our team has looked extensively at issues related to the treatment of BPSD in Virginia nursing homes through projects funded both by our home institution (VCU) and the Virginia Center on Aging [13-15,31-34,92]. In Virginia, nursing home providers have increased their prescribing of mood-stabilizing AEDs as they have decreased their use of antipsychotics, benzodiazepines, and other monitored psychotropics. Long-stay antiepileptic prescribing is increasing even though long-stay reporting of the diagnosis of seizure epilepsy is decreasing [13-15,31-33,93]. Similar to what has been described in European nursing homes, the increases in mood-stabilizing AED use is concentrated in the long-stay subgroup of residents with dementia and without a diagnosis of epilepsy [26]. In fact, more mood-stabilizing antiepileptics are now prescribed to the subset of Virginia nursing home residents without a diagnosis of seizure epilepsy than antipsychotics are prescribed to the entire nursing home population [13].

Significant differences also exist in AED prescribing for residents with epilepsy compared with those without. Mood-stabilizing AEDs dominate prescribing for residents without an epilepsy diagnosis, whereas non–mood-stabilizing AEDs are the preferred therapy for residents with epilepsy [13,15,90]. Also relevant, since 2012, Virginia nursing home providers have increased use of the exclusionary diagnoses (primarily schizophrenia) that allow for antipsychotic prescribing without mandatory reporting to CMS quality measures via the MDS, likely inflating apparent reductions in inappropriate antipsychotic use [32,33]. We believe that this diagnostic trend represents another unintended consequence of reduction initiatives.

The variance between long-stay facilities in the top and bottom quintiles for mood stabilizer prescribing in Virginia is also significant. Facilities in the top quintile for mood stabilizer use in Virginia had significantly more male and African American residents and were more likely to be privately owned. Facilities in the bottom quintile for mood stabilizer use had vastly better scores for social determinants of health, including higher health opportunity indexes with superior health opportunity, economic opportunity, consumer opportunity, environmental, and wellness disparity rankings [34]. Similarly to all risky medications, prescribing of mood-stabilizing AEDs is more common in vulnerable populations. In Virginia, both antipsychotic and antiepileptic mood stabilizer prescribing is more common in male African American individuals [13,34]. Parallel qualitative assessment from our mixed methods pilots added supporting context as long-stay prescribers were frank that sensitivity to CMS reporting and treatment guidelines emphasizing antipsychotic deprescribing influenced both documentation and management choices [15,31,92].

We are only just beginning to see all the impacts of the COVID-19 pandemic play out in measurable nursing home outcomes. We already know that adverse health events such as potentially avoidable hospitalizations are associated with breakdowns in communication and care processes as well as with shortages in staffing and resources [53]. We know that facilities have struggled to provide adequate staffing during the pandemic for many reasons. Anecdotally, resident loneliness and negative mood symptoms spiked dramatically as the pandemic stressed facilities already underpowered to manage BPSD using NPT. In Virginia, we have seen significant deterioration in MDS measures for functional independence; an increased need for activities of daily living support; worsening of detrimental behavioral symptoms; declining mood scores; and escalated prescribing of all psychoactive classes, predominantly an acceleration of the trend toward unmonitored AEDs [93].

This proposal addresses specific and clinically meaningful evidence gaps directly relevant to clinical guidelines and policies and deeply consequential to all stakeholders in dementia care, including residents, providers, specialty groups, payers, patient advocates, and policy makers. If pharmacological approaches to BPSD management rapidly evolved during the COVID-19 pandemic, with risky and ineffective—but unreported—medications being used more as medications mandatorily reported to the CMS are used less, then harms could be increasing with no clinical or safety benefit, jeopardizing the health and safety of all nursing home residents but of vulnerable populations most of all. In addition, we know almost nothing about outcomes or adverse health events associated with this changing approach to dementia care. Our proposed study will close this existing knowledge gap by providing a clearer picture of all long-stay pharmacological approaches to noncognitive dementia symptoms, the health outcomes associated with these varying approaches, and how such management and consequent outcomes are changing under the pressure of pandemic stressors and targeted medication reduction efforts.

### Strengths and Limitations

This study is not without limitations. While the comprehensiveness of our sample, which includes Medicare or Medicaid residents from all US states, is an asset, our sample is limited to include only those with continuous Fee-For-Service, Medicare Advantage, and Part D coverage. This means that residents with a Part D alternative or without a drug plan will be excluded. However, the coverage of nursing home residents is more stable than that of noninstitutionalized populations, and we know that our study will include the vast majority of long-stay residents [94]. We also view our long 12-year study period as a strength and, given our complex models, we are minimizing the potential for overfitting by using the maximal number of time points available from the MDS (48). While our study will assess for the presence of FDA-approved indications for specific drugs, a step toward approximating the appropriateness of medication use, we acknowledge that FDA-approved indications comprise only a fraction of reasonable prescribing. Unfortunately, we do not believe that guidelines are congruent enough that we could easily add guideline-driven indications to this investigation. This study will identify and describe meaningful trends in long-stay prescribing and outcomes. Still, we acknowledge that many potential confounders exist that impact management approaches, and definitive explanations of the root cause of these trends will need to be addressed in future evaluations. We will perform sensitivity analyses to evaluate secular trends in our data. In addition, our proposed survey will add nuance and context that can help explain the trends that we discover. Coping with data inconsistencies when linking data sets is always a challenge. However, we are comfortable with methods that have resulted in matching errors of <1% to 4% [48,49]. Finally, although the effort and resources required for a representative national survey would be excessive to realize aim 3, we can still accomplish a purposive sample that can approximate what is known about the long-stay prescriber population and is sufficient to provide a meaningful understanding of the forces behind prescribing decisions [95,96].

### Conclusions, Dissemination, and Future Directions

Drugs with unproven efficacy and concerning risk profiles continue to be overused for BPSD, although purposeful reduction efforts are curbing antipsychotic and anxiolytic prescribing. However, pilot data suggest that, while use of these targeted drugs is decreasing, off-label prescribing of antiepileptics is increasing. Unlike antipsychotics, anxiolytics, and other psychotropic medications used for BPSD, antiepileptic use in nursing homes is not mandatorily reported to the CMS via the MDS, and even basic national prevalence data about their long-stay use are lacking. Moreover, nothing is known about potentially adverse health outcomes related to these prescribing changes. If pharmacological approaches to BPSD management are evolving, with medications not reported to the CMS being used more and medications reported to the CMS being used less—all with completely unknown patient-oriented outcomes—this has an impact on clinical guidelines and policies and direct relevance to all stakeholders in dementia care, including residents, providers, specialty groups, payers, patient advocates, and policy makers. This study will detail the use of all psychotropics and the outcomes associated with their use and evaluate changes and trends in prescribing and in outcomes with an emphasis on the impact of policies and the COVID-19 pandemic.

We view a complete detailing of all pharmacological approaches to BPSD as a critical first step to optimizing dementia care in nursing homes. Describing the patient-oriented outcomes associated with all such prescribing is an equally important step 2. Closing these knowledge gaps and providing all stakeholders with a clearer perspective is crucial to informing ongoing policies, guidelines, and improvement measures. Reaching multiple stakeholder groups will be facilitated by our multidisciplinary team as dissemination will in turn also be multidisciplinary, with geriatric nurses, nurse practitioners, anthropologists, epidemiologists, primary care and long-term care physicians, pharmacists, public health, and social workers all represented. The dissemination process will involve sharing our data with all our diverse partner organizations. Our team has detailed experience with the traditional formats of dissemination, including social media, blogs, commentaries, and peer-reviewed publications and conference presentations. We view this project as an essential stepping stone in an ongoing line of inquiry. Our overarching goal is to improve dementia care by ensuring that, if drugs are needed, the right medications are used at the right dose, at the right time, and as a last resort. Related to that, identifying and deconstructing barriers to NPT for BPSD as well as providing clinicians and families with more precise, helpful, and evidence-based BPSD decision tools are ongoing team goals.

## Abbreviations

AD: Alzheimer disease
ADRD: Alzheimer disease–related dementias
AED: antiepileptic drug
BPSD: behavioral and psychological symptoms of dementia
CMS: Centers for Medicare and Medicaid Services
DUA: Data Use Agreement
ED: emergency department
FDA: Food and Drug Administration
IRB: Institutional Review Board
MDS: Minimum Data Set
MedPAR: Medicare Provider Analysis and Review
NH: nursing home
NPT: nonpharmacologic therapy
PPIN: Psychotropic Prescribing in Nursing Homes
RIF: Research Identifiable File
VCU: Virginia Commonwealth University
VRDC: Virtual Research Data Center

## Data Availability

Data sharing is not applicable to this article as no data have been collected and the data set has not yet been generated or analyzed. Once data becomes available, the data sets generated during and analyzed during this study will be available from the corresponding author on reasonable request.
